# Twelfth Annual Meeting of the American Medical Association

**Published:** 1859-06

**Authors:** 


*Twelfth Annual Meeting of the American Medical Association.*— We publish in full, from the "*Louisville Journal*," the proceedings of this august body, possessing, at this time, some features which are both novel and interesting. After eleven years, the subject of medical education, which has been one of the important ends and aims of the Association, as regards its improvement, since its first session, has assumed a new form, promising as protracted and unsatisfactory an existence as that with which the profession is now so familiar. This variegation is in the shape of a Convention of Medical Teachers, meeting the day before the regular Convention, the proceedings of which we also publish. The value of this movement will be appreciated on the perusal of its proceedings. As yet, the only progress which has been made is an adjournment for one year, an appointment of a committee of five to take *certain matters* into consideration, and the appointment of a Committee of Conference from the Association itself.

In the Association we notice nothing especially new, excepting the change in time of meeting from May to June, and the want of time for examining the prize essays, preventing the award of a prize for this year, which should admonish competitors in future to send in their productions early. We sincerely congratulate our venerated preceptor and friend, Prof. Miller, on the honor which the Association has conferred upon him. No one could have been selected who would impart more dignity to the position, or who, by his professional and private character, is more worthy of it.

### MEDICAL TEACHERS' CONVENTION.

LOUISVILLE, May 2, 1859.

If the medical fraternity could always carry about such delicious weather as this; if, like the Norwegian saga, they could bottle up the balmy vernal breezes and prescribe them in ultra-allopathic doses to the invalid, what life-bearing remedies would always be at their disposal, and how the pharmacopœa would be simplified! The Convention of Medical Teachers was called under the following resolution, adopted at the eleventh annual meeting of the American Medical Association, held at Washington city, last year:

*Resolved*, That we recommend to all the medical colleges entitled to a representation in this body, that they appoint delegates especially instructed to represent them in a meeting to be held at Louisville on Monday, the day immediately preceding the Convention of the American Medical Association for the year 1859, at ten o'clock in the morning, at such place as the committee of arrangement shall designate.

In pursuance of this resolution the committee selected Mozart Hall for the place of meeting, and they made every provision for the comfort of the delegates and the gentlemen of the press, each of the latter having a convenient table with requisite stationery and a most luxurious arm chair prescribed for his accommodation. This evidence of attention deserves our warmest acknowledgments.

At the hour of 10, the Convention was called to order, and Prof. Dixi Crosby, of Dartmouth College, Hanover, N. H., was selected as Chairman, and Prof. George C. Blackman, of Ohio Medical College, at Cincinnati, as Secretary. Prof. Crosby, on assuming the chair, said that, like all his predecessors called upon to preside over deliberative bodies, he had been taken wholly by surprise, and should have declined had not Dr. Frost, of S. C., and Dr. Davis, previously excused themselves from serving. He could bring the Convention no qualification for the position except an earnest desire to serve them; but this and the support of the members he hoped would enable him to meet their approval and conduct the important deliberations satisfactorily.

Rev. J. H. Haywood was then introduced, and invoked the Divine supervision over the proceedings of the body in an earnest and eloquent prayer.

Some discussion then ensued as to the mode of organization, some wishing all medical professors present to act as delegates, and others desiring that each college should have a unit representation. The following resolution was submitted by Dr. David F. Wright, of Shelby Medical College:

*Resolved*, That all members of the Faculties of Medical Colleges now present shall be considered members of this Convention, but that where more than one belong to the same College, one of them alone shall vote in behalf of that institution.

After some further interchange of views—all tending to the same wish of full representation—on motion of Dr. A. H. Baker, of Cincinnati, the following substitute was offered and adopted :

*Resolved*, That a committee of three on credentials be appointed by the chair.

Under this resolution, Prof. Crosby selected Drs. Baker, Shattuck, and Haskins the Committee on Credentials, and the Convention took half an hour's recess for the registration of the names of delegates.

But the Committee on Credentials have discharged their duty ; the Convention is called to order again, and the following delegates are announced as in attendance, with the institutions to which they belong :

*Dartmouth College, N. H.*, Professor Dixi Crosby ; *Shelby Medical College*, Prof. E. B. Hastings, Prof. D. F. Wright ; *Missouri Medical College*, Prof. J. N. McDowell ; *St. Louis Medical College*, Prof. M. L. Linton ; *Medical College of S. C.*, Prof. Henry K. Frost ; *Medical College of Ga. at Augusta*, Prof. H. F. Campbell, Prof. Joseph Jones ; *Medical Department University of Michigan*, Prof. Moses Gunn ; *University of Louisville*, Prof. L. P. Yandell, Prof. L. Powell ; *Cincinnati College of Medicine*, Prof. A. H. Baker ; *Lind University, Chicago*, Prof. N. S. Davis ; *Oglethorpe Medical College*, Prof. A. G. Thomas ; *Medical College of Ohio*, Prof. George C. Blackman ; *Western Reserve Medical College, Cleveland, O.*, Prof. G. C. C. Weber ; *Kentucky School of Medicine*, Prof. M. Goldsmith, Prof. G. W. Bayless ; *Iowa University*, Prof. McGugin ; *Medical College of Memphis, Tenn.*, Prof. H. R. Robards ; *Medical College of Richmond, Va.*, Prof. B. R. Welford, Prof. L. L. Joynes ; *Atlanta Medical College, Ga.*, Prof. J. G. Westmoreland, Prof. John W. Jones ; *Medical Faculty of Harvard University, Boston, Mass.*, Prof. Geo. C. Shattuck ; *Rush Medical College, Chicago, Ill.*, Prof. Daniel Brainard, Prof. Joseph W. Freer.

The Convention was then permanently organized by the reëlection of the temporary officers, Prof. Crosby humorously remarking that the delegates were fortunate in this action, inasmuch as they would have no further speech in reference to the honor conferred, &c. He said that, until the Convention should adopt rules for its government, he should limit speeches to ten minutes, and allow no one to speak more than twice on the same subject without permission.

Dr. Wright's resolution that members from Medical Colleges who are now present be permitted to take part in the debates, but that each college have but one vote, was again taken up, considered and passed.

Dr. N. S. Davis offered the following, which was adopted :

" *Resolved*, That a business committee of five be appointed by the chair to report propositions for the action of the convention."

The chair appointed Drs. N. S. Davis, Gunn, Frost, Shattuck and Yandell. After a short recess to enable this committee to report, they submitted the following, through Dr. Davis, the chairman :

"1. *Resolved,* That this convention recognizes the great advantages to be derived from the action of the American Medical Association in prescribing the terms and conditions on which medical degrees shall be conferred and licenses to practice medicine shall be granted; and that an expression of opinion as to methods or periods of instruction from the American Medical Association should be received with deference and respect, and that all pains should be taken to enforce any rules and regulations recommended by that body:

"2. *Resolved,* That this convention earnestly recommend the American Medical Association to adopt such measures as will secure the efficient practical enforcement of the standard of preliminary education adopted at its organization in May, 1847; and that the medical colleges will cheerfully receive and record the certificates alluded to in said standards, whenever the profession generally and the preceptors will see that the students are properly supplied with them.

"3. *Resolved,* That no medical college should allow any term of practice to be a substitute for one course of lectures in the requisitions for graduation.

"4. *Resolved,* That Hospital Clinical Instruction constitutes a necessary part of medical education; and that every candidate for the degree of Doctor of Medicine should be required to have attended such instruction regularly for a period of not less than five months during the last year of his period of medical pupilage.

"5. *Resolved,* That every medical college should rigidly enforce the rule requiring three full years of medical study before graduation, and that the diploma of no medical college shall be recognized which is known to violate this rule."

Prof. Wright, of Nashville, moved that the resolutions of the report be considered seriatim; and the first being taken up, he spoke at length in opposition to it, giving a history of the previous difficulties between the American Medical Association and the medical colleges. He could neither vote for such a resolution, nor could he take any future part in the proceedings of a convention which should adopt it.

Prof. Brainard, of Chicago, thought this convention was asked to take a step fraught with peril to the harmony of the profession and its best interests; it should be met on the threshold, and a solemn protest entered against it. This body did not represent the medical colleges of the country with unanimity; New York, Philadelphia and New Orleans are not represented here, and he must consider their absence as a protest against the assumption of any power on the part of this body or the American Medical Association to dictate the terms on which the colleges should confer their degrees or receive their students.

The admission of such a resolution would produce hostile factions both in the profession and in the colleges, and could never receive the sanction of those who had independent, chartered rights to fall back upon. He was opposed to no true improvement in the medical profession, but he did object to shutting that door upon young men desirous of entering the profession, through which we ourselves all had entered.

Without definite action on the resolution, the convention adjourned until 3 o'clock P. M.

AFTERNOON SESSION.

When the convention reässembled, Dr. Bayless offered the following amendments to the first resolution :

"1. To substitute in the third line the word 'recommending' for 'prescribing.'

"2. To strike out all after the words 'deference and respect.'"

A long discussion ensued on the resolution, which was participated in by Doctors Bayless, Yandell, Palmer, McDowell, Davis, Brainard, Shattuck, Baker and Wright. The differences of opinions seemed almost as various as the number of speeches, and the convention was tying itself into an apparently inextricable entanglement, when an Alexander sprung up in the person of Prof. L. L. Joynes, of the Medical College of Richmond, Va., who offered the following preamble and resolutions as a substitute for the resolutions from the Business Committee :

"Whereas, It appears that a large proportion of the medical colleges of the United States are unrepresented in this convention, and no changes in the present system of education can be effectual unless adopted by the schools generally—

"*Resolved*, That it is inexpedient at this time to take any action upon the proposition contained in the report presented by the Special Committee on Medical Education, at the last meeting of the American Medical Association.

"*Resolved*, That with the view of obtaining a more general union in council and in action upon this important subject, this convention do now adjourn, to meet again on the day preceding the next annual meeting of the American Medical Association, at the place which may be agreed upon for said meeting, and that the several medical colleges in the United States be requested to appoint each one delegate to such adjourned meeting of this convention."

These resolutions were amended, at the suggestion of Dr. Wright, to include the appointment of a committee of five to take into consideration, during the recess, the various matters referred to in the resolutions, and to report thereon at the adjourned meeting.

The vote was demanded on this by colleges, and resulted as follows :

Yeas—Shelby Medical College, Missouri Medical College, St. Louis Medical College, Oglethorpe Medical College, Ohio Medical College, Western Reserve Medical College, Kentucky School of Medicine, Medical College, Richmond, Atlanta Medical College, Rush Medical College—10.

Nays—Medical College, S. C., Medical College, Ga., Medical Department University Mich., University of Louisville, Cincinnati College of Medicine, Lind University, Iowa University, Medical College, Memphis, Harvard University—9.

The substitute was declared adopted—yeas 10, nays 9—and so the convention stood adjourned until the day preceding the next annual meeting of the American Medical Association.

The chairman appointed the following committee under the above resolution : Drs. Yandell, Shattuck, Blackman, Campbell and Gunn.

TWELFTH ANNUAL MEETING OF THE AMERICAN MEDICAL ASSOCIATION.

Louisville, May 3, 1859.

The association met at 11 o'clock A. M., in Mozart Hall, the President,

Dr. Harvey Lindsay, of the district of Columbia, in the chair, supported by Drs. W. L. Sutton, of Kentucky, Thomas O. Edward, of Iowa, Josiah Crosby, of Massachusetts, and W. C. Warren, of North Carolina, as Vice-Presidents, with Drs. Alexander J. Semmes, of the district of Columbia, and S. M. Bemiss, of Kentucky, acting as Secretaries. Dr. Caspar Wistar, of Penn., Treasurer, was also in attendance.

The President announced the Rev. Mr. Robinson, of Louisville, who opened the proceedings with prayer.

Dr. Robert J. Breckinridge, chairman of the committee of arrangements, then welcomed the delegates to the city.

Prof. Joshua B. Flint, of Louisville, accompanied by Drs. Sutton, Chipley, Spillman, and Snead, then came forward and addressed the President as follows:

"Mr. President:—At a late annual meeting of the 'State Medical Society of Kentucky,' the following resolution was unanimously adopted, and the gentlemen before you, all of them ex-Presidents of the Society, constituted a committee charged with carrying it into effect:

"'*Resolved*, That ——— be a committee to wait upon the A. M. Association, so soon as it shall have opened its session in Louisville, and in behalf of this society bid it welcome to the medical jurisdiction of Kentucky, assure it of the cordial interest of the profession of the State in the objects and purposes of its institution, and of the readiness of this Society to coöperate in all its endeavors to promote the honor and usefulness of our common calling.'

"In regard to assurances of welcome, Mr. President, so far as they apply to yourself and your associates as individual guests of your Kentucky brethren, those gentlemen would hardly pardon me for adding a word to the general terms of the resolution. Already, if I mistake not, there are demonstrations of the spirit of hospitality, which render any assurances on that subject worse than superfluous.

"But I am happy to assure you, Mr. President, that the Association over which you preside, in its corporate capacity, with its well-known purposes and ends, will find an equally cordial reception in the general community which it has now honored with its presence. The people of Kentucky, sir, are generally supposed to appreciate, as it deserves, every enterprise of a public-spirited or philanthropic character which presents itself to their notice, and I think I may say, especially disposed to befriend the cause of Medical Education. They have certainly done somewhat a little to their credit in evidence of their intelligent interest in Medical Science and the best means of its advancement. Through the munificence of the State, in one case, and of this liberal city in the other, two medical libraries have been procured in Kentucky, each of which is superior to any and all the public collections of medical books that can be found in most of the other States of the Union. Not more than two of our sister States, so far as I can learn, can be compared with us in this interesting particular.

"One of these libraries, belonging to the Medical Department of the University of Louisville as its best estate, numbering 4,000 volumes, you will doubtless visit during your sojourn among us; and although much defaced and mutilated by the conflagration which laid that institution in ruins two years ago, you will still find it to be a large and choice collection—adequate to the requisitions of medical research, and representing satisfactorily the course of medical literature from the time of Hippocrates to the present day.

"The other library to which I refer belongs to the Medical Department of Transylvania University, and contains 8,000 volumes. I hope that not a few of the members of the Association, before leaving Kentucky, will find their way into that also, in the course of a visit to the beautiful inland city in which it is located—a city distinguished throughout the land for the general intelligence and refinement of its population, as well as for the eminent public men who have signalized it as their home; but to medical men, not only of our own, but of foreign countries, especially memorable as the residence of the great lithotomist of our day, and surgical patriarch of the West—Benjamin W. Dudley.

"Such benefactions as these to the means of medical study attest, as I have already intimated, so enlightened an interest in the improvement of our profession as to guarantee not only a welcome to the Association which represents it, but efficient coöperation in its endeavors on the part of the profession and people of Kentucky.

"May your present session, Mr. President, be an agreeable one to the members of the Association, and prove eminently beneficial to the interests of American medicine."

The Secretary, Dr. Bemiss, then called the roll of members of the Association, and the following gentlemen were in attendance: * * * *

The president then appointed the following gentlemen a committee on voluntary essays: Drs. L. P. Yandell of Kentucky, Bryan of Philadelphia, and Comegys of Ohio.

Dr. R. J. Breckinridge, from the committee of arrangements, announced the hours of business from 9 A. M. to 12 M., and from 3 P. M. until such hour as the convention should adjourn upon resolution, which arrangement was adopted.

Dr. Harvey Lindsly, the President of the Association, then read his retiring address, which was listened to with marked attention, and was an eloquent tribute to the dignity of the medical profession and the importance of its improvements.

After he had concluded, Dr. L. A. Smith, of New Jersey, moved that the thanks of the Association be tendered to the president for his able and eloquent address, and it was ordered to be placed in the hands of the appropriate committee, for publication among the proceedings of the meeting.

Dr. Caspar Wistar, chairman of the committee on publication, read the annual report, and on motion of Dr. Sayre, of New York, the following resolutions appended to it were unanimously adopted:

*Resolved*, That hereafter every paper intended for publication in the Transactions must not only be placed in the hands of the Committee of Publication by the 1st June, but it must also be so prepared as to require no material alteration or addition at the hands of the author.

*Resolved*, That authors of papers be required to return their proofs within two weeks after their reception,—otherwise they will be passed over and omitted from the volume.

Adjourned until 3 o'clock P. M.

AFTERNOON SESSION.

Dr. W. L. Sutton, one of the Vice Presidents, took the chair in the absence of the President.

Dr. D. Meredith Reese, of New York, chairman of the Committee on Nominations, reported the following officers for the ensuing year:

President—Henry Miller, of Kentucky.

Vice Presidents—H. F. Askew, Delaware; Charles S. Tripler, U. S. Army; L. A. Smith, New Jersey; Calvin West, Indiana.

Treasurer—Caspar Wistar, Pennsylvania.

Secretary—S. M. Beemis, Kentucky.

Dr. Sayre moved the adoption of the report, which was unanimously agreed to.

Dr. Brainard of Illinois, moved the appointment of a committee to conduct the newly appointed officers to their respective chairs. The acting president selected Drs. Brainard of Illinois, Mattingly of Kentucky, Sutton of Indiana, McDowell of Mo., and R. J. Breckinridge of Kentucky, and they accordingly performed the duties assigned to them.

The newly elected President, on taking the chair, addressed the Convention in substance as follows:

*Gentlemen of the American Medical Association:* I am wholly at a loss to command language to express the deep sense of obligation put upon me by calling me to the Presidency of your Association. It is an honor any man may well be proud of, and although I admit, in all sincerity, that you might without difficulty have selected an individual more worthy the position, I may be allowed to say you could not have conferred it upon one who would prize it more highly, or cherish it longer with the most grateful recollections. I do esteem it the greatest honor ever conferred upon me by the profession that I love and to which I have devoted a long life; nay, more, it is the greatest honor that could be conferred upon any man by the medical or any other profession in this or any other country; for any decoration of honor or any mark of approbation conferred by a crowned head I should regard as a bauble in comparison. Who are you, gentlemen, when rightly considered? You are the rightful representatives of the great American Medical Profession —an army forty thousand strong, and a body of men, no matter what captious criticism may say in disparaging comparison with the European branch of the profession, in my humble judgment, far superior to the same number of medical men to be found in any quarter of the globe. Although as a body you may not be so learned, so critically and nicely framed in all the minutiæ of the profession, yet for strength, integrity, and precision in all the great principles guiding to a successful combat with disease, this body is equal if not superior to that of any kingdom of continental Europe.

To be called to the Presidency of such a body of men, is in my sober judgment the greatest compliment that could be conferred on mortal man, provided that man is a devotee of medicine, who has given his whole mind, soul, heart and strength individually to the profession, and has that high regard for it which will not suffer any less noble pursuit to interfere with the daily, though laborious duties of the profession. Coming so recently from a sick-bed, and still enfeebled in health, I beg to be excused from further remarks, and desire you to accept this brief and imperfect acknowledgment of the distinguished honor conferred upon me, instead of what, under other circumstances, I might be disposed to say.

The President, after this graceful address, sat down amid much applause, when Dr. R. J. Breckinridge moved that the thanks of the Association be tendered to the retiring officers for the faithful and assiduous manner in which they have conducted the business committed to their charge, which was unanimously adopted.

A long and discursive debate then ensued on the admission of members by invitation. The plan of organization permits practitioners of respectable standing, from sections of the United States not otherwise represented at the meeting, to receive appointment by invitation of the meeting, after an introduction from any of the members present, or any absent permanent members, to hold connection with the Association until the close of the annual session at which they are received, and to be entitled to participate in all its affairs as in the case of delegates. The point of difficulty seemed to be, whether the invitations should be extended by the Committee of Arrangements or by open vote of the Association. It was finally settled by referring all the applicants' names to the Committee on Arrangements.

Dr. J. B. Lindsly, of Tennessee, offered the following:

*Resolved,* That a committee of three be appointed by the chair to inquire into and report upon the propriety of dividing the Association into sections for the purpose of performing such parts of its scientific labors as may relate to particular branches of medicine and surgery.

Dr. Brodie moved its reference to the Nominating Committee.

Dr. Brainard explained at some length the object of the resolution of inquiry, and enforced its adoption as a means of giving more effect and usefulness to the proceedings of the Association, the reports of which had heretofore gone out unmatured, in consequence of the want of concentrated action.

A motion by Dr. Sayre to lay the motion on the table was negatived, and the motion of Dr. Lindsly was then adopted.

Dr. Davis moved that no person be permitted to speak more than twice on the same subject, or more than ten minutes at one time, except by consent of the Association, which was adopted.

The Standing Committee on Prize Essays was called on for their report, but without a response. This was also the case with the Committee on Medical Education. The Committee on Medical Literature had no report to present.

A letter from Dr. J. G. F. Holston, of Ohio, Chairman of the Special Committee on the Microscope, was read, reporting progress and begging a continuance for more extended investigation, which was referred to the Committee on Nominations.

A letter from Dr. Stephen Smith, of New York, from the Special Committee on Medical Jurisprudence, had the same reference.

The Special Committee on Quarantine was not ready to report.

Dr. Mattingly, of Ky., from the Special Committee on Diseases and Mortality of Boarding-Schools, asked a continuance until next year, in order to obtain further information requisite to the full investigation of the important subject. The request was referred to the Committee on Nominations.

The Special Committees on Surgical Operations for the relief of defective vision, on milk sickness, and on the blood corpuscle, had the same reference.

A report from the Committee on Medical Ethics, signed by Dr. John Watson, of New York, was read, laid on the table, and made the special order for to-morrow at 12 o'clock M. This is an important subject, and will probably give rise to much debate to-day. We publish the report in full, as follows:

TO THE AMERICAN MEDICAL ASSOCIATION.

The Committee on Medical Ethics beg leave to state that, of the subjects referred to them at the last meeting of the association, they find the following notice in the minutes:

"Dr. Grant, of New Jersey, presented a complaint made by the Newark Medical Society against the New York Medical College, for a violation of the ethics of the profession. Dr. Edwards, of Iowa, presented a similar complaint; and Dr. Oakley, of New Jersey, a complaint from the Union and Essex County Medical Society."

*Transactions, Vol. XI., p.* 41.

Upon these several complaints your Committee beg leave most respectfully to report:

That the two complaints from the Medical Societies of New Jersey refer only to one and the same grievance, the particulars of which are set forth in a memorial which was presented to the American Medical Association on the 6th of May, 1848, and which is entitled: "Statement of the Newark Medical Association in Reference to a Diploma Granted by the New York Medical College."

The facts stated in this memorial which is now appended to this report, were, during the last annual meeting of the American Medical Association, examined as carefully as time and opportunity would allow. The charges therein contained against the New York Medical College were admitted to be true by Dr. Horace Green, President of said College, who, in apology for the same, submitted a written statement to your Committee, which was at the time accepted as satisfactory by the gentlemen then present before your Committee on behalf of the parties aggrieved; and being afterwards presented with a verbal report by the Committee, was received and entered upon the minutes in the following terms:

"*Whereas,* It appears from undoubted testimony that the New York Medical College have conferred the degree of Doctor of Medicine upon a notorious quack of the name of John F. Dunker, of Newark, the Faculty, in the person of the President of said College, wish here to declare, that the degree was obtained under gross deception and false testimonials furnished by said Dunker and his friends; and they therefore revoke and annul his diploma, and declare said Dunker to be unworthy of patronage or support from authority conferred upon him by this diploma."

*Transactions, Vol. XI., p.* —.

These complaints being thus disposed of, your Committee have only to add in reference to them, that the memorial presented to the American Medical Association from the Newark Medical Association is worthy of special notice, as setting forth the negligent manner in which mere verbal and hearsay statements are at times accepted in place of authentic written testimonials, from individuals presenting themselves as candidates for the honors of our profession at some of the Medical Colleges of this country. In this respect there is reason to believe that the New York Medical College does not stand alone, and the publication of the accompanying memorial may be of service in putting a permanent check to this crying evil.

The only other complaint referred to your Committee was that presented by Dr. Edwards, of Iowa, preferring a charge from the Dubuque Medical Society against one of her members who had been expelled for an alleged infraction of the code of medical ethics. This complaint does not appear to be of such a character as to require adjudication here. It has, since the last annual meeting of the American Medical Association, been adjudged by the Iowa State Medical Society, (see transactions of the annual meeting of said Society, published at Dubuque, Iowa, 1858), and having been then settled in the State in which the parties reside, it should now be dismissed.

All of which is respectfully submitted,

JOHN WATSON, M. D., Chairman.

New York, April 28, 1859.

Continuances were asked by the committee on the Pons Varrolii, Medulla Oblongata, and Spinal Marrow—their Pathology and Therapeutics; on American Medical Necrology; on the Hygienic relations of Air, Food, and Water, the Natural and Artificial Causes of their Impurity, and the best Methods by which they can be made most Effectually to contribute to the Public Health; on the Effect of the Virus of Rattlesnakes, &c., when Introduced into the System of the Mammalia; on the Climate of the Pacific Coast and its Modifying Influences upon Inflammatory Action and Diseases generally; on the Constitutional Origin of Local Diseases, and the Local Origin of Constitutional Diseases; on the Physiological effects of the Hydro-Carbons; on Epilepsy; on the causes of the Impulse of the Heart, and the Agencies which influence it in Health and Disease, and on the best Substitutes for Cinchona, and its Preparations in the Treatment of Intermittent Fever, &c., all of which were referred to the Committee on Nominations.

The Special Committee on Government Meteorological Reports made a report, written by Dr. R. H. Coolidge, of the U. S. Army, but read by Dr. Paul F. Eve, of Tennessee, which was referred to the Committee on Publications.

The Committee, appointed in May, 1857, on Criminal Abortion, submitted a report, written by Dr. Storer, of Boston, which was read by Dr. Blatchford, of New York, and referred to the Committee on Publication. The following resolutions appended to this report were unanimously adopted:

*Resolved*, That while physicians have long been united in condemning the act of producing abortion, at every period of gestation, except as necessary for preserving the life of either mother or child, it has become the duty of this Association, in view of the prevalence and increasing frequency of the crime, publicly to enter an earnest and solemn protest against such unwarrantable destruction of human life.

*Resolved*, That in pursuance of the grand and noble calling we profess—the saving of human lives—and of the sacred responsibilities thereby devolving upon us, the Association present this subject to the attention of the several Legislative Assemblies of the Union with the prayer that the laws by which the crime of procuring abortion is attempted to be controlled may be revised, and that such other action may be taken in the premises as they in their wisdom may deem necessary.

*Resolved,* That the Association request the zealous coöperation of the various State Medical Societies in pressing the subject upon the Legislatures of their respective States, and that the President and Secretary of the Association are hereby authorized to carry out by memorial these resolutions.

The Convention then adjourned till to-morrow morning at 9 o'clock.

SECOND DAY'S PROCEEDINGS.

WEDNESDAY, May 4, 1859.

The President, Dr. Miller, called the Association to order at 9 o'clock.

Dr. D. Meredith Reese, chairman of the Committee on Nominations, called attention to the fact that the committee could not act definitely until the place for next year's meeting should be designated. He stated also that the Medical State Society of Connecticut had requested that an amendment to the constitution proposed two years since should be taken from the table, relative to the time of meeting.

It was moved by Dr. Blatchford, and seconded by Dr. Sayre, that the amendment to the third article of the constitution be taken up, which proposes to add after the words "first Tuesday of May" the words "or first Tuesday of June," and after the words "shall be determined" add the words "with the time of meeting."

The amendment was adopted by a constitutional vote.

Dr. D. M. Reese also stated that the Connecticut State Society had extended a pressing invitation to the Association to hold its next meeting at New Haven, which invitation was referred to the Committee on Nominations.

Dr. Reese also called attention to the necessity of some radical change in the mode of appointing committees to prepare treatises on scientific subjects to be reported at the annual meetings. It had been seen that, on yesterday, a large majority of the committees made no reports, and did not even see proper to send in any communication explanatory of delay. The difficulty heretofore has originated in the mode of selection adopted by the nominating committee. It has been customary for gentlemen to hand in their names and the proposed subjects on slips of paper, and the committee, without further investigation, have so published in the annual reports. Thus it has happened that appointments have been most injudiciously made, and gentlemen to whom a special duty has been assigned, have been found to know less of that than any other subject. He therefore hoped that no committee of last year would be reäppointed or continued, from which no report had been had and no communication received.

On motion, the nominating committee was unanimously instructed to act upon the suggestions of the chairman, who also stated that there should be some definite expression of disapprobation as to the course of those gentlemen who had volunteered essays, and had their names reported in the newspapers and spread over the land, and then paid no further attention to the matter.

Dr. Flint, from the Committee on Prize Essays, begged leave to report that they received four dissertations in time for a careful and thorough examination, and two others, quite voluminous, only two days before the meeting of the Association. The latter we have felt constrained to exclude altogether from the competition of the present year, on account of the absolute impossibility of reading them with a critical purpose and effect. The others have been carefully examined by all the surviving members of the committee—one estimable associate, Dr. Evans, having been called from all his earthly labors before the active duties of the committee began.

More than one of the four essays we examined exhibited much labor, and a commendable scholarship in their preparation—are voluminous, and in some respects very meritorious papers; but, in the unanimous judgment of the committee, neither of them possess the degree and species of merit which should entitled its author to the association prize.

The committee beg leave furthermore to report that, in their opinion and as the suggestion of their own recent experience, the association should determine in more precise and formal manner than has yet been done, the terms and conditions of competition and of success in the contest for prizes, for the government alike of contestants and the committee of adjudication, and that a committee be now appointed to consider and report upon that subject.

Dr. J. B. Lindsly, chairman of the Committee appointed to inquire into the propriety of dividing the Association into sections, for the better performance of its work in considering the various branches of medicine and surgery, recommended the adoption of such a plan as being indispensably necessary to making this body a working scientific association. They do not deem it necessary to enter into any argument in favor of this plan, it being the one already universally adopted by similar bodies. They would simply recommend, for the present, a division into the following sections, as being most suitable to facilitate the transaction of business, viz:

1. Anatomy and Physiology.
2. Chemistry and Materia Medica.
3. Practical Medicine and Obstetrics.
4. Surgery.

The committee do not propose that this subdivision of labor shall in any manner interfere with the regular business of the Association as now conducted, but only that after having assembled each day in general session, each section shall meet separately for the purpose of hearing and discussing papers on such subjects as properly belong to them; and they therefore recommend that the Committee of Arrangements for the ensuing year be requested to provide suitable accommodations for the services of these sections, and that each of said sections shall be authorized to make such arrangements as may be required for the proper transaction of its business.

This report was considered and adopted, after a very able speech in its support by Dr. Davis.

Dr. J. W. Singleton, of Ky., moved the suspension of the rules for the introduction of the following:—

*Resolved,* That in the death of Dr. A. Evans, of Kentucky, the Association has lost one of its most manly and efficient members, and society a friend and benefactor.

The resolution was unanimously adopted.

Dr. W. L. Sutton, under the resolution appointing a Committee on registration of births, marriages, &c., proposed a plan of general action, an abstract of which he read, on motion of Dr. Gibbs, of S. C.; and on motion of Dr. L. P. Yandell, the subject was referred to a committee, to report during the present session.

Drs. Sutton, Lindsly, W. R. Gibbs, Bryan, Pitcher and Crosby were appointed such committee.

Dr. Blatchford stated that he had received from Dr. Willard, Secretary of the New York State Medical Society, fifty volumes of their Transactions for 1859, for distribution to the medical press, the medical colleges, and all medical societies of the South, and sent with a request for an interchange of civilities. Gentlemen present can be supplied by application to Dr. Bemiss, and if the number sent be not sufficient for the supply, they will be cheerfully forwarded to any gentleman, by application to the Secretary, Dr. S. D. Willard, Albany, N. Y., the postage being included in the application, which is twenty-two cents.

A voluminous report from Dr. Thomas Logan, of California, on Medical Topography and Epidemics, was received and referred to the Committee on Publications.

The chairman of the Committee on Voluntary Essays stated that he had received a paper on a case of extra-uterine fœtation from Dr. Enos Hoyt, of Transylvania, Mass., and another on a case of accidental poisoning by strychnine, from Dr. Douglas Bly, of Rochester, N. Y. He also presented a very voluminous paper entitled, "Observations on some of the changes of the Solids and Fluids in Malarial Fever, by Joseph Jones, Prof. of Medical Chemistry in the Medical College of Georgia, at Augusta." By request, Prof. Jones gave a verbal abstract of his paper and an exposition of his theory, and on motion of D. W. Yandell the communication was referred to the Committee on Publications.

Dr. D. W. Yandell announced that the following railroad companies had agreed to pass delegates to this convention over their roads at half price; Pittsburg, Fort Wayne, and Chicago; Pennsylvania Central; Jeffersonville; New Albany and Salem; Louisville and Nashville, and Cleveland and Pittsburg.

On motion, a vote of thanks was tendered to these companies for their liberality.

Dr. J. B. Flint offered the following resolution:

*Whereas,* Our brethren of Great Britain are engaged in erecting a monument to the memory of John Hunter, whose invaluable services in behalf of Physiology and Surgery are recognized and honored, as well on this side of the Atlantic as in Europe; and whereas, this Association, as the representatives of American Medicine, would rejoice in some suitable manner to participate in so grateful a testimonial of gratitude and respect; therefore—

*Resolved,* That a committee of three be appointed to consider in what manner this participation can best be effected, so as to be acceptable to our British brethren, and consistent with our own means and opportunities of action, with instructions to report at the next annual meeting.

The resolution was adopted, and Drs. Flint, Bowditch, and Shattuck appointed as the committee.

Dr. Harvey Lindsly offered the following :

*Whereas*, Parliamentary rules of order are numerous, complicated, sometimes obscure, and often inapplicable to such a body as the American Medical Association ; and whereas, from the nature of the pursuits of medical men, they cannot be familiar with these rules ; therefore,

*Resolved*, That a select committee of three members be appointed to prepare a system of rules for the government of this Association, as few in number, as concise and as perspicuous as possible, to be reported to the next annual meeting.

This resolution was adopted, and Drs. Lindsly, Comegys and Blatchford appointed as the committee.

The paper of Dr. Bly, on Accidental Poisoning by Strychnine, was read by its author, and as individual cases are not reported in the journals of the Association, thanks were returned for the communication, with a request that it be published in some medical journal.

An invitation from Grand Master Morris, of the Masons, was received, urging medical brethren to attend the Masonic Convention now in session in this city.

The Nominating Committee made the following report :

The next annual meeting to take place at New Haven, on the first Tuesday of June, 1860. Dr. Eli Ives is elected junior secretary.

*Committee of Arrangements*, Drs. Chas. Hooker, Stephen G. Hubbard, and Benjamin Sullivan, Jr., with power to add to their numbers ; *Committee on Prize Essays*, Drs. Worthington Hooker, Conn., G. C. Shattuck, Mass., Usher Parsons, R. I., P. A. Jewett, Conn., and John Knight, Conn. ; *Committee on Publication*, Drs. F. G. Smith, Philadelphia, Pa., Wistar, do., Bemiss, Louisville, Ky., Ives, New Haven, Conn., Hollingsworth and Hartshorne, Philadelphia, Pa., and Askew, Wilmington, Del. ; *Committee on Medical Literature*, Drs. Henry Campbell, Ga., D. F. Wright, Tenn., O. Wendell Holmes, Mass., S. G. Ormer, Ohio, and W. H. Byford, Ill. ; *Committee on Medical Education*, Drs. D. M. Reese, N. Y., W. R. Bowling, Tenn., Chas. Fishback, Ind., John Bell, Penn., Z. Pitcher, Mich.

The following Special Committees were appointed :

*On Morbus, Coxarius, and Surgical Pathology of Articular Inflammation*, Dr. Lewis A. Sayre, of New York ; *On the Surgical Treatment of Strictures of the Urethra*, Dr. James Bryan, of Philadelphia ; *On Drainage and Sewerage of Large Cities, their Influence on Public Health*, Drs. A. J. Simmes, D. C., chairman, Cornelius Boyle, and G. M. Dove ; *On the Periodicity of Diseases Prevailing in the Mississippi Valley*, Dr. J. W. Singleton, of Smithland, Ky. ; *On Puerperal Tetanus, its Statistics, Pathology, and Treatment*, Dr. D. L. McGugin, of Keokuk, Iowa ; *On Hospital Epidemics*, Dr. R. K. Smith, of Philadelphia ; *On Puerperal Fever*, Dr. J. N. Green, of Stelisville, Ind. ; *On Anœmia and Chlorosis*, Dr. H. P. Ayres, of Fort Wayne, Ind. ; *On Veratrum Viride*, Dr. James B. McCraw, of Richmond, Va. ; *On Alco-*

*hol, Its Therapeutical Effects*, Dr. J. R. W. Dunbar, of Baltimore, Md.; *On Meteorology*, Dr. J. G. Westmoreland, Atlanta, Ga.; *On Milk Sickness*, Dr. Robert Thompson, Columbus, Ohio; *On Manifestations of Disease of Nerve Centres*, Dr. C. B. Chapman, Wisconsin; *On the Medical Topography of Iowa*, Dr. T. O. Edwards, Iowa.; *On Microscopic Observations on Cancer Cells*, Dr. Geo. D. Norris, New Market, Ala.; *On the Philosophy of Practical Medicine*, Dr. James Graham, Cincinnati, Ohio; *On Some of the Peculiarities of the North Pacific, and their relations to Climate*, Dr. Wm. H. Doughty, Ga.

The following Special Committees were continued or altered:

*On Microscope*, John C. Dalton, Jr., N. Y., David Hutchinson, Ind., A. R. Stout, Cal., Calvin Ellis, Mass., Christopher Johnson, Md.; *On Diseases and Mortality of Boarding Schools*, Dr. C. Mattingly, Ky., and Dixi Crosby, N. H.; *On the Various Surgical Operations for the Relief of Defective Vision*, Drs. M. A. Pullen, Mo., T. J. Cogley, Ind., and W. Hunt, Penn.; *On the Blood Corpuscle*, Dr. A. Sayer, Michigan; *On American Medical Necrology*, Dr. C. C. Cox, Maryland; *On the Hygienic Relations of Air, Food, and Water*, the natural and artificial causes of their impurity, and the best methods by which they can be made most effectually to contribute to the public health, Dr. C. C. Cox, Md.; *On the Effect of Virus of Rattlesnake, &c.*, when introduced into the system of Mammalia, Dr. A. S. Payne, Virginia; *On the Climate of the Pacific Coast*, and its Modifying Influences upon Inflammatory Action and Diseases generally, Dr. O. Harvey, Cal.; *On the Constitutional Origin of Local Diseases* and the Local Origin of Constitutional Diseases, Drs. W. H. McKee, North Carolina, and C. F. Heywood, New York.

On motion of Dr. Brodie, Dr. A. J. Semmes was requested to serve as Secretary pro tem. during the remainder of the session.

The Association took up the special order, being the report on Medical Ethics, to which had been referred the action of the Dubuque Medical Society, which, after debate, was laid over until 12 o'clock to-morrow.

On motion of Dr. H. F. Campbell, a section of meteorology, medical topography, and epidemic diseases, and of medical jurisprudence and hygiene, was added to those already adopted by this association.

Amendments to the constitution of the association were then taken up, and a provision was acted upon that no individual who shall be under sentence of expulsion or suspension from any State or local medical society, of which he may have been a member, shall be received as a delegate to this body, or be allowed any of the privileges of a member until he shall have been relieved from such sentence by such State or local society. This amendment to the constitution was adopted.

The next amendment, lying over from last year, was the proposition of Dr. Kyle, of Ohio.

That the constitution of the association be so amended as to prohibit the admission as a delegate, or the recognition as a member, of any person who is not a graduate of some respectable Medical College.

This amendment was rejected; but, on the question of reconsideration, a long and animated debate ensued, which called forth all the oratorical abilities and much of the personal feelings of the delegates. Without arriving at a vote, the association adjourned for dinner.

AFTERNOON SESSION.

Upon the reässembling of the Association, the discussion was renewed on the motion to reconsider the vote by which the amendment to the constitution was negatived, prohibiting the admission as a delegate, or the recognition as a member, of any person who is not a graduate of some respectable Medical College.

Dr. Kincaid moved a further amendment, to insert the word "hereafter" after "prohibiting."

Dr. Askew of Delaware, one of the Vice Presidents in the chair, ruled the amendment out of order at the present stage, or until the Association decide upon the question of reconsideration.

After a long discussion, Dr. Davis, of Indiana, moved to lay the motion to reconsider on the table, which was carried, 97 yeas, nays not counted ; so the amendment stands registered.

The next proposed amendment, to the constitution was that suggested by the New Jersey Medical Society, asking for such changes as would establish a Board of Censors in every judicial district of the Supreme Court, who should examine and grant diplomas to all proper members of the association.

This was temporarily laid on the table for Dr. Crosby to offer a report of the Medical Teachers' Convention, which met on Monday last. He strongly recommended a committee from this body to confer with the Teachers' Committee, and felt great confidence that something beneficial to medical education would be the effect of such conference.

Dr. Comegys moved the appointment of a committee of five to confer with the committee of Medical Teachers and report at the next annual meeting, provided that no medical teacher be selected on the part of this association.

This again gave rise to an excited debate, clearly showing that there was a great deal of bad feeling between the Professors and the laymen of the profession. Professor McDowell, of Missouri, was extremely happy in some of his hits, and kept his auditors in a roar of laughter. He acknowledged that Philadelphia and New York had the advantage of location ; the railroads took students there as they did the horses and cattle of the West, and sometimes its asses.

Professor Crosby, of Dartmouth College, contended that the elevation of the standard of medical education depended more upon practitioners than the colleges ; if bad materials were sent up from physicians' offices for Professors to model into physicians, it could not be expected that good results would follow. He wanted a committee of conference, not based on any sectional feelings, and he believed the whole matter could be arranged satisfactorily.

Dr. D. W. Yandell wished to reply to one remark of Professor Crosby, as to the bad materials sent by private teachers to the colleges. He had himself rejected students who were too big fools to be made physicians, and these same persons, in a few months, had gone to some of the colleges and come back with their diplomas in their pockets. After a very eloquent, appropriate, and conciliatory speech from Dr. Davis, the resolution from Dr. Comegys was unanimously adopted.

The resolutions from the New Jersey Medical Society were then taken from the table and referred to the Committee of Conference.

Dr. Davis offered a resolution instructing the same committee to confer with the State Medical Societies for the purpose of procuring more decisive and uniform action throughout the profession, in carrying into effect the standard of preliminary education adopted by this Association at its organization in 1847. This was carried.

D. Gibbes, from the committee to examine into a plan of uniform registration of Births, Marriages, and Deaths, offered the following report:

They have given the same a careful consideration, and they unanimously recommend that the Report be adopted and referred to the Committee on Publication.

They also recommend that the same committee be continued, with instructions to add to the Report in time for publication in the ensuing volume of Transactions a form of registration law which may be likely to answer the requirements of the several States.

Dr. Sayre, of N. Y., offered the following:

*Whereas*, The medical profession at large have an interest in the character and qualifications of those who are to be admitted as their associates in the profession—therefore,

*Resolved*, That each State Medical Society be requested to appoint annually two delegates for each college in that State, whose duty it shall be to attend the examination of all candidates for graduation, and that the colleges be requested to permit such delegates to participate in the examination, and vote on the qualifications of all such candidates.

This was referred to the Committee of Conference.

The paper of Dr. Jones, presented at the morning session, was taken from the committee on Publication and referred to the committee on Prize Essays.

Dr. Eve moved to record the name of Dr. Benjamin W. Dudley as a permanent member, which was adopted by a unanimous vote, the delegates all rising to their feet in token of respect.

Adjourned till to-morrow morning.

The following members of the Association registered their names during the day: * * * *

The whole number of delegates in attendance is therefore 301, exclusive of members by invitation.

### THIRD DAY'S PROCEEDINGS.

THURSDAY, May 5, 1859.

The President called the Association to order at 9 o'clock, and the reading of the minutes of yesterday was dispensed with.

The first business in order was an amendment to the constitution, laid over from last year, and proposed by Dr. T. L. Mason, of New York, to insert in the first line of the second paragraph of Article 2, after the words "shall receive the appointment from," the words "any medical society permanently organized in accordance with the laws regulating the practice of physic and surgery in the State in which they are situated, and consisting of physicians and surgeons regularly authorized to practice their profession." Also to add to the sixth paragraph of the same article the words, "but each permanent member of the first class designated in this plan of organization shall be entitled to a seat in the Association on his presenting to this body a certificate of his good standing, signed by the secretary of the society to which he may belong at the time of each annual meeting of this body."

Dr. Linden A. Smith, of New Jersey, said amendments to the constitution should be adopted with care, and though, perhaps, that now proposed might be desirable, still, as Dr. Mason, who had proposed it, was not present to explain his views, he moved that the subject be laid over until next year. This suggestion was adopted.

Another constitutional amendment, proposed by Dr. Henry Hartshorne, of Penn., and laid over from last year, under the rules, provides to add to the 2d article the words, "No one expelled from this Association shall at any time thereafter be received as a delegate or member, unless by a three-fourths vote of the members present at the meeting to which he is sent, or at which he is proposed."

This amendment was adopted.

Another amendment, proposed by J. Berrien Lindsley, of Tenn., was called up, to omit in article 2 the words "medical colleges, hospitals, lunatic asylums, and other permanently organized medical institutions in good standing in the United States," and also to omit the words, "The faculty of every regularly constituted medical college or chartered school of medicine shall have the privilege of sending two delegates. The professional staff of every chartered or municipal hospital, containing a hundred inmates or more, shall have the privilege of sending two delegates, and every other permanently organized medical institution of good standing, shall have the privilege of sending one delegate."

This was laid on the table until the next annual meeting.

An invitation was received from Mons. Groux, requesting the delegates to meet him at the hall of the University at noon to-day, to witness experiments on his congenital fissure of the sternum, which was deferred until 4 o'clock this afternoon, as the association had previously accepted the hospitality of Mr. and Mrs. Robert J. Ward at the former hour.

Dr. Singleton offered a series of resolutions from "Young Physic," deprecating the introduction of schisms, and reflecting on the harmony of the Association, which, after a vigorous speech from Dr. McDowell, was unanimously laid on the table, with a request that it should not appear on the minutes. Dr. Davis regarded the evidences of harmony and good feeling exhibited here this year as greater and more cheering than on any previous occasion, and deprecated any insinuations that unkindly sentiments existed.

Dr. McDermot submitted the following resolutions:

"*Whereas*, A vast proportion of the disease and misery that afflict our race is caused by the excessive use of intoxicating liquors; and whereas, in the opinion of this Association, the evils of intoxication can be most effectually remedied by the establishment of Inebriate Asylums, wherein the victims of intemperance may be subjected to such restraints and treatment as shall effect a thorough reformation of their habits; therefore—

"*Resolved*, That this Association recommend the establishment of Inebriate Asylums in the various States of the Union.

"*Resolved*, That the State and County Medical Societies, and all members of the medical profession, be requested to unite in diffusing among the people a better knowledge and appreciation of the beneficent purposes and important benefits that would be conferred upon Society by the establishment of such asylums throughout the various sections of the country."

This resolution was referred to the mover as a special committee, with a request that he would report thereon at the next meeting of the Association.

Dr. Shattuck offered the following, which was adopted—

*Resolved*, That the committee appointed in May, 1857, on Criminal Abortion, be requested to continue their labors, and especially to take all measures necessary to carry into effect the resolutions reported by them on the first day of the meeting.

Dr. Yandell, from the Committee on Voluntary Essays, made a further report, that a communication had been received from Dr. Langer, of Iowa, on Subentaneous Injections as remedials, which, on motion, the author read.

The essay was referred to the writer as a special committee, with the request that he would report further at the next annual meeting of the Association, and continue his investigations.

Invitations to visit the Insane Asylum and the Library and Museum of Transylvania University were received.

The president appointed as the committee of conference to meet the committee from the Teachers' convention, the following gentlemen: Dr. Blatchford, Troy, N. Y.; Condie, Philadelphia, Pa.; Bozeman, Montgomery, Ala.; Brodie, Detroit, Michigan; and Sneed, Frankfort, Ky.

Dr. D. Meredith Reese, from the nominating committee, made the following final report:

Special committees continued.

*On Quarantine*—Drs. D. D. Clark, Penn.; Snow, R. I.; Jewell, Penn.; Fenner, La., and Houck, Md.; *On Medical Ethics*—Drs. Schuck, Penn.; Murphy, Ohio; Linton, Mo.; Powell, Ga.; Eve, Tenn.; *On Tracheotomy in Membranous Croup*—Dr. A. V. Dougherty, N. J.; *On the Effects of Lithotomy*, Performed in Childhood, upon the Sexual Organs in After Life—Dr. White, Memphis, Tenn.; *On Mercurial Fumigation in Syphilis*—Dr. D. W. Yandell, Louisville, Ky.; *On the Improvement in the Science and Art of Surgery*, made during the last Half Century —Dr. Jos. McDowell, St. Louis, Mo.; *On the Cause and Increase of Crime*, and its mode of Punishment—Dr. W. C. Sneed, Frankfort, Ky.; *On the Education of Imbecile and Idiotic Children*—Dr. H. P. Ayres, Fort Wayne, Ind.; *On the Uses and Abuses of the Speculum Uteri*—Dr. C. H. Spillman, of Kentucky.; *On the Topography of Vermont*—Dr. Perkins, of Vermont.; *On the Pons Varolii, &c.*—Dr. S. B. Richardson, of Kentucky, and Dr. Fishback, of Indiana; *On the Physical Effects of the Hydro-Carbons*—Dr. F. W. White, of Illinois; *On the Effect of the Periodical Operations for Urinary Calculi upon Procreation in the Male*—By J. S. White, of Tennessee.

The paper from Dr. Ellis, of Massachusetts, on the subject, "Does the microscope enable us to make a positive diagnosis of cancer, and what if any are the sources of error?" was referred to the special committee on the microscope, of which Dr. Dalton is chairman.

Honorary resolutions were passed to the memory of the following members of the Association, deceased: Dr. W. W. Bowling, of Alabama, Dr. Thomas D. Mutter, of Penn.; Dr. P. C. Gaillard, of S. C.; Dr. Jabez G. Goble, of New Jersey; Dr. John K. Mitchell, of Penn.

Dr. R. K. Smith, of Philadelphia, submitted the following:

*Resolved*, That the death of Dr. John K. Mitchell, one of the members of this Association, has been to this body a loss keenly felt by every man who knew him. His eminence as a teacher, his varied acquirements in every department of learning, and his generous social qualities in every relation, endeared him to every member of the profession who had the pleasure of his personal acquaintance.

*Resolved*, That the family be notified of the action of this Association.

Other more formal resolutions were offered and feeling eulogies pronounced.

Dr. Sayre offered the following, which were adopted by acclamation:

*Resolved*, That the thanks of the American Medical Association are eminently due and are hereby presented to the citizens of Louisville, Ky., for the princely hospitality publicly and privately extended to the members of this body during its present session.

*Resolved*, That to the Committee of Arrangements and the profession of Louisville generally, our thanks are due for their kind and assiduous attention to the Association, and for the hearty welcome with which they have greeted our convention in their flourishing city.

After the transaction of some other unimportant routine business,

On motion of Dr. Davis, the Association adjourned, to meet at New Haven on the first Tuesday in June, 1860.

The registration book during the day announced the names of Drs. D. G. Thomas, of New York; William S. Cain, of Kentucky, and Peter Allen, R. K. McMeans, and W. R. Kable, of Ohio—making 305 members in attendance during the session of the Association.

